# *ANGPT1* methylation and delayed cerebral ischemia in aneurysmal subarachnoid hemorrhage patients

**DOI:** 10.1186/s43682-021-00001-7

**Published:** 2021-12-20

**Authors:** Dongjing Liu, Annie I. Arockiaraj, John R. Shaffer, Samuel M. Poloyac, Paula R. Sherwood, Sheila A. Alexander, Elizabeth A. Crago, Daniel E. Weeks, Yvette P. Conley

**Affiliations:** 1 Department of Genetics and Genomic Sciences, Icahn School of Medicine at Mount Sinai, New York, NY, USA.; 2 Department of Human Genetics, Graduate School of Public Health, University of Pittsburgh, Pittsburgh, PA, USA.; 3 Department of Oral Biology, School of Dental Medicine, University of Pittsburgh, Pittsburgh, PA, USA.; 4 Department of Pharmaceutical Sciences, School of Pharmacy, University of Pittsburgh, Pittsburgh, PA, USA.; 5 School of Nursing, University of Pittsburgh, 440 Victoria Building, 3500 Victoria Street, Pittsburgh, PA 15261, USA.; 6 Department of Biostatistics, Graduate School of Public Health, University of Pittsburgh, Pittsburgh, PA, USA.

**Keywords:** Aneurysmal subarachnoid hemorrhage, Delayed cerebral ischemia, Epigenome-wide association study, Methylation, *ANGPT1*

## Abstract

**Background:**

Delayed cerebral ischemia (DCI) is a common secondary complication and an important cause of disability and mortality among patients who survive aneurysmal subarachnoid hemorrhage (aSAH). Knowledge on DCI pathogenesis, risk factors, and biomarkers are essential for early detection and improved prognosis. To investigate the role of DNA methylation in DCI risk, we conducted an epigenome-wide association study (EWAS) in 68 patients followed up to 1 year after the initial aneurysm rupture. Blood samples were collected within 48 h post hemorrhage and used for DNA methylation profiling at ~ 450k CpG sites. A separate cohort of 175 patients was sequenced for the top CpG sites from the discovery analysis for a replication of the EWAS findings.

**Results:**

EWAS did not identify any epigenome-wide significant CpGs. The top signal, cg18031596, was annotated to *ANGPT1*, a gene with critical functions in angiogenesis after vascular injury. Post hoc power calculations indicated a well-powered discovery analysis for cg18031596. Analysis of the replication cohort showed that four out of the five CpG sites sequenced at the *ANGPT1* locus passed a Bonferroni-adjusted significance threshold. In a pooled analysis of the entire sample, three out of five yielded a significant *p*-value, and the top association signal (*p*-value = 0.004) was seen for a CpG that was not originally measured in the discovery EWAS. However, four *ANGPT1* CpG sites had an opposite effect direction in the replication analysis compared to the discovery EWAS, marking a failure of replication. We carefully examined this observed flip in directions and propose several possible explanations in addition to that it was a random chance that *ANGPT1* ranked at the top in the discovery EWAS.

**Conclusions:**

We failed to demonstrate a significant and consistent effect of *ANGPT1* methylation in DCI risk in two cohorts. Though the replication attempt to weaken the overall support of this gene, given its relevant function and top rank of significance in the EWAS, our results call for future studies of larger aSAH cohorts to determine its relevance for the occurrence of DCI.

## Introduction

Delayed cerebral ischemia (DCI) is a common secondary complication among aneurysmal subarachnoid hemorrhage (aSAH) patients who survived the initial bleeding caused by ruptured aneurysm. It represents a significant yet potentially preventable cause of mortality and poor functional outcomes. The knowledge of the pathophysiology of DCI has evolved over years and it is thought that a combination of multiple mechanisms underlies DCI and poor functional outcomes, including cerebral vascular dysregulation, microthrombosis, cortical spreading depolarization, and neuroinflammation [1]. The complex process involves a number of different tissues and cell types, for example the smooth muscle cells in microvessels, the endothelia at the blood-brain barrier, and the resident immune cell microglia of the central nervous system [2]. Some recognized potential risk factors for DCI are delayed treatment, female gender, smoking, and alcohol use [1]. Previous research efforts toward the identification of genetic factors underlying DCI have focused on a few candidate genes [3]. These studies presented inconclusive results and so far very few genome-wide searches have been conducted.

Epigenetics provides a new perspective in studying the etiology of human diseases. Epigenome-wide association studies (EWASs) have substantially enhanced our knowledge of a variety of human disorders and provided valuable insights into their molecular mechanisms [4]. Because the human epigenome is sensitive to environmental stimuli, brain injury caused by aneurysm rupture can potentially trigger changes in the methylation profiles at relevant genomic sites, which may influence pathophysiological processes thereafter. The epigenetic changes associated with DCI pathogenesis has not been extensively characterized; so far only a few small studies reported a limited number of genes whose methylation levels may be associated with DCI, including *INSR, CDDH5, ITPR3*, and *HAMP* [5–7]. Most studies examined blood methylation profiles, which although they may be inferior to those from brain tissues, can still be useful as blood cells are informative of the inflammatory responses triggered by the DCI event [8, 9].

The goal of this study was to investigate the role of DNA methylation in DCI occurrence in aSAH patients, using a two-stage study design (Fig S1). In the discovery stage, we conducted an EWAS which tested whether DNA methylation profiles characterized in the peripheral blood of 68 aSAH patients are associated with the risk of DCI. In the replication stage, we followed up the top EWAS signal in a larger patient cohort using targeted bisulfite sequencing. We also investigated whether the associated CpGs can predict long-term recovery outcomes of the patients.

## Material and methods

### Discovery subject recruitment

Participants were considered for this study if they were admitted to the University of Pittsburgh Medical Center (UPMC) Neurovascular Intensive Care Unit with an aSAH confirmed by cerebral angiography and a Fisher grade (hemorrhage burden) > 1, aged between 21 and 75, and had external ventricular drain placed as standard of care to drain cerebrospinal fluid. SAH due to arteriovenous malformation, trauma, or mycotic aneurysm was excluded. Blood samples were collected from 88 aSAH patients chosen in a way that DCI cases and controls were roughly balanced for the sake of optimal power in downstream analysis. To rule out the possibility that methylation changes were a consequence of DCI occurrence, only 68 samples collected within 48 h from the initial bleeding event and prior to DCI occurrence were qualified for further analysis. Demographics collected include age, sex, race, smoking status, height, and weight. Patients were followed during the acute phase for DCI and vasospasm occurrence, as well as during a long-term outcome phase up to 1 year to assess the Glasgow Outcome Scale (GOS) and Modified Ranking Scores (MRS). Informed consent was obtained from the participant or their legal proxy using a protocol approved by the University of Pittsburgh Institutional Review Board.

### Discovery sample methylation quality control

The quality control process was detailed in a previous study [10]. In short, genome-wide methylation levels in the blood samples for the discovery cohort were quantified using Illumina Infinium HumanMethylation450 Beadchip at the Genetic Resources Core Facility SNP Center of Johns Hopkins University. All samples were placed on a single plate, and cases and controls for DCI were balanced within chips using a checkerboard pattern to guard against the possibility of DCI status being confounded with row, column, or chip effects. We carried out a variety of quality checks and filters including the detection of outliers and low-quality samples, background correction, dye bias correction, functional normalization, and CpG-level quality filters. Specifically, we performed functional normalization [11] to reduce noise and technical variation due to batch effects, which also increased concordance between technical replicates [12]. Table S1 detailed the number of probes removed by each CpG-level quality filter, including the removal of probes overlapping SNP, probes on sex chromosome, cross-reactive probes, probes exhibiting multi-modal distributions indicative of poor quality, and probes that were inadequately detected in more than 1% of samples. After the quality control procedure, a total of 418,247 CpG sites were retained. As it has been proposed that *M*-values exhibit more favorable statistical properties compared with beta values [13], we used *M* values for further statistical analysis.

### EWAS

We fitted a linear regression model with the empirical Bayes moderation in the EWAS analysis, using the eBayes function in the limma R package {Ritchie: 2015fa}. In this regression model, the methylation *M* value was regressed on the DCI status while adjusting for age and sex. As methylomic profiles differ widely by cell types, modeling cell type heterogeneity across samples is crucial in any methylation association studies using blood samples. We therefore included surrogate variables obtained from surrogate variable analysis (SVA) as additional covariates to remove the effect of cell type heterogeneity and uncontrolled sources of variation in our samples [14]. As suggested by Saffari et al. [15], the threshold for declaring epigenome-wide significance was set at 2.4 × 10^−7^. In order to identify differentially methylated regions (DMRs) where DNA methylation at multiple CpG sites are consistently associated with DCI, we used the dmrff R package developed by Suderman et al. [16].

### Association with recovery outcomes

To examine the effect of methylation at top EWAS hits on long-term recovery, we fitted a linear model regressing the *M* value on the recovery outcomes while adjusting for age, sex, and surrogate variables. Two types of recovery measurements were collected: GOS and MRS measured at 3 months (GOS-3 and MRS-3) and 12 months (GOS-12 and MRS-12) post-discharge. The GOS and MRS were dichotomized as either favorable (GOS 4 to 5; MRS 0 to 1) or unfavorable (GOS 1 to 3; MRS 2 to 6). Generally, patients with favorable outcomes may have minor deficits but not severe disability and they need not rely on caretakers for normal life. Patients with unfavorable outcomes were either dead, in vegetative state, or severely disabled and were dependent on others. Fatality at the same time points (Death-3 and Death-12) was derived from GOS/MRS (the most severe category denotes a death outcome).

### Replication cohort and methylation profiling

The replication cohort (*N* = 175) was recruited together with the discovery cohort, i.e., at the same clinic, using the same inclusion criteria and spanning roughly the same time period, except that they were not selected to be assayed on the Illumina 450k Beadchip in the initial discovery EWAS. Phenotype data collection procedures were identical to those used in the discovery cohort. Bisulfite methylation sequencing using pyrosequencing (referred to as MethylSeq hereafter) was used to quantify the methylation levels at a targeted genomic region from DNA extracted from blood samples. Bisulfite conversion was done following standard protocols with the Epitect Bisulfite kit (Qiagen, Inc.). Converted samples were PCR’d using Qiagen Pyromark PCR Kit (PN: 978703), following standard protocols. The assay (CG18031596, PN: PMC0087707), was custom designed by Qiagen. Sequencing was done on aPyromark Q48 Autoprep nstrument using recommended reagents (PN 974002 & 947203) following the instrument protocol (firmware v.4.03, software v.4.2.1, OS v.1.1.2). Data was called using the Pyromark Q48 Autoprep 2.4.2 software. The probe sequence was designed to cover from 10 bp downstream to 65 bp upstream of the EWAS top CpG cg18031596 (Supplemental Methods). This captured cg18031596 in addition to four additional variable CpG sites in this region. Pyrosequencing has internal bisulfite conversion controls built into the sequence and samples with incomplete conversion were not used for sequencing. Samples that failed the Methyl-Seq assay at a certain site were excluded from analyses of that site. A subset of the discovery samples included in the EWAS (*N* = 58) were also re-assayed by MethylSeq along with the replication samples, bringing the total number of subjects with available MethylSeq data to *N* = 233.

### Replication association analysis

Methylation calls at each CpG site were initially generated as a beta value and were then logit transformed into an *M* value. We fitted a linear regression model predicting *M* value at each CpG sites as a function of the DCI status/recovery outcomes while adjusting for age and sex. To account for multipletesting, we implemented the procedure based on eigenvalues proposed by Li and Ji [17] and calculated the effective number of tests among the five CpGs to be 1.1. We therefore used a Bonferroni threshold of 0.05/1.1 = 0.045 to declare significant association in the replication stage. Because a subset of the discovery samples was re-assayed by MethylSeq which generated new data that has not been analyzed at the point of the study, our replication stage included analyzing both the discovery and the replication samples, first separately and then altogether. With only 5 CpG sites measured, we were not able to construct and adjust for surrogate variables in the replication stage. To evaluate the potential influence of cell type heterogeneity on the replication results, we re-used the surrogate variables generated in the discovery stage for the 58 re-assayed discovery samples (referred to as “proxy surrogate variables” thereafter), and compared the adjusted results to the main replication (unadjusted) results.

### Simulation

As noted in the “Results” section below, we observed a reversed association direction in the replication analysis compared to the EWAS results. To explore how often this could have occurred by chance in a smaller discovery sample and a larger replication sample, we carried out a simulation study designed to mirror the workflow of the real analysis, on the top EWAS CpG site cg18031596. Specifically, the entire sample (233 discovery and replication samples who have available MethylSeq data) was randomly divided into a discovery group and a replication group, each of the same size as the real discovery/replication cohort (58/175). These two groups were then analyzed in the same way as we did for the real data. To mimic the small *p* values observed in the EWAS (which led us to pursue a replication), we only retained the simulated discovery/replication duos where the discovery *p*-value was less than 0.05, and recorded the regression coefficient of cg18031596 in these duos. We repeated the random split 10,000 times, from which the distributions of discovery regression coefficients and replication regression coefficients were generated and compared, and the chance of observing effects of opposite direction was quantified.

## Results

### Discovery EWAS

Participant characteristics are summarized in Table 1. Samples from 68 participants passed the quality control criteria for the 450K array methylation data. This patient population had a mean age of 53.7 years (range 31 to 74) and the majority were females (71%). The sample population was largely White (86%). Roughly half of the patients suffered DCI within the acute follow-up period. DCI cases and controls were similar in mean age (52.0 and 54.6), female proportion (71.4% and 69.7%), and proportion of smokers (58.1% and 58.6%).

**Fig. 1:**
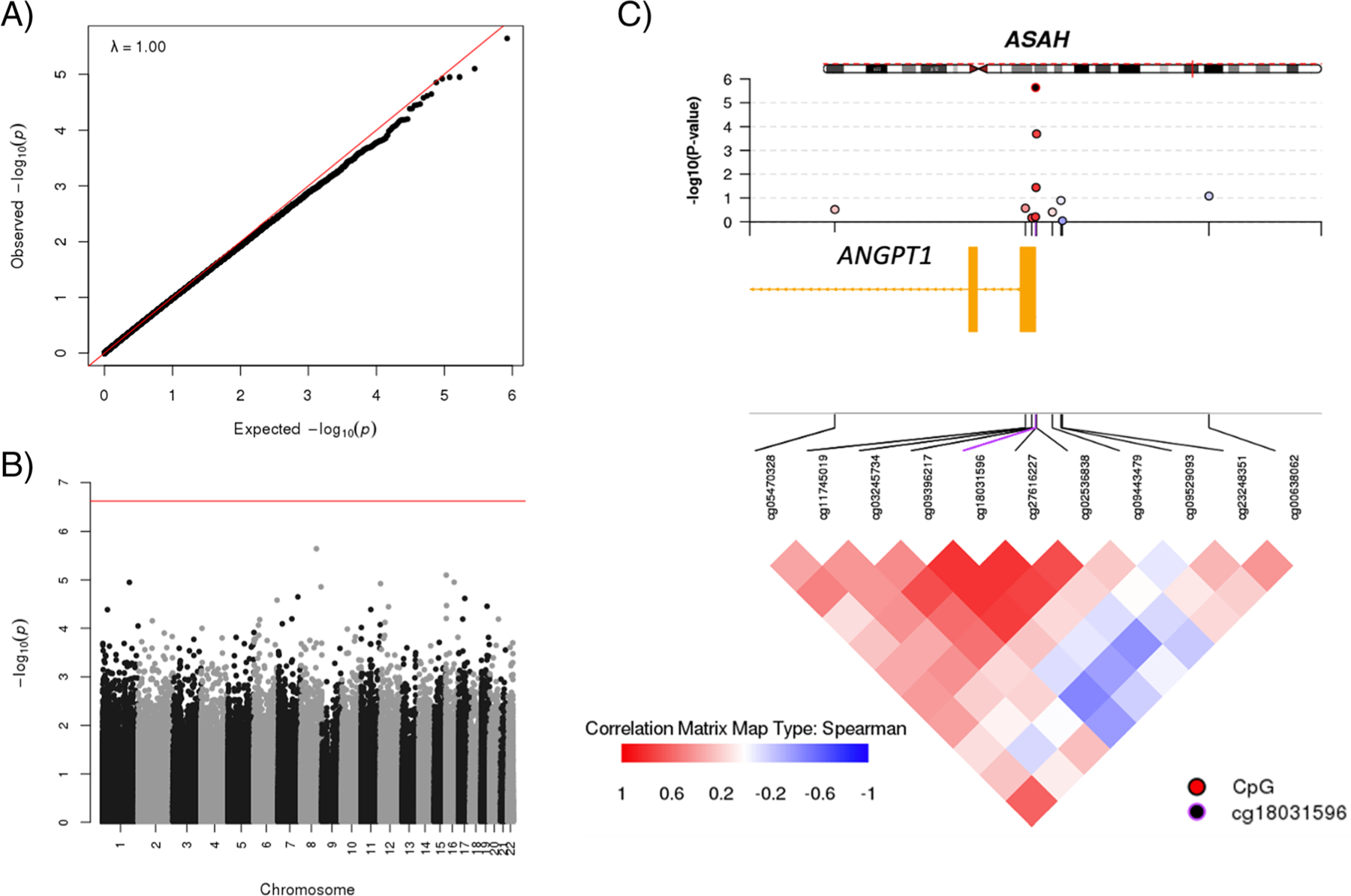
EWAS of DCI. **A** Quantile-Quantile plot showing expected (*x*-axis) and observed (*y*-axis) -log10-transformed *p*-values. **B** Manhattan plot showing the -log10-transformed *p*-values (*y*-axis) for each CpG by its genomic position (*x*-axis) organized by chromosome. **C** coMET plot for the *ANGPT1* locus. For the coMET plot, the upper panel shows the EWAS *p*-values; the middle yellow track represents the gene position with exons indicated by boxes; the lower panel shows the correlation between selected CpGs in this genomic region

There was no sign of inflation in the EWAS statistics (Fig. 1). None of the CpG tested surpassed the epigenome-wide significance level of 2.4 × 10^−7^. The most significant signal occurred at cg18031596 which was annotated to the Angiopoietin gene 1 (*ANGPT1*) on chromosome 8, with a *p*-value of 2.3 × 10^−6^ and a Bayesian factor of 2.4 (Table S2). The mean methylation beta value at this site was 2.20% and 1.79% in cases and controls, respectively. The use of surrogate variables for the control of unwanted variation strongly influenced the significance level of the cg18031596 signal (cg18031596 *p*-value = 0.05 without adjustment for surrogate variables). In the genome-wide search for DMRs, the same site, cg18031596, again displayed the strongest signal with a *p*-value = 3.3 × 10^−7^, despite it being a single-CpG region. We further explored whether this CpG was associated with long-term recovery outcomes, and found that a lower M value at cg18031596 was associated with better GOS-3 (*p* = 0.006) and had a borderline association (*p* = 0.047) with GOS-12 (Table S3).

We performed a post hoc power calculation for cg18031596 using the online EPIC Array Power Calculator [18]. In the 68 discovery samples, the mean relative difference in the *M* value of cg18031596 in DCI cases and controls was ~ 5%. We had 94.6% power to detect a true 5% mean difference or larger at a significance threshold of *p*-value =1 × 10^−7^, indicating a well-powered discovery analysis for this particular CpG.

### Replication results

Although the *p*-value at the *ANGPT1* CpG cg18031596 did not reach the epigenome-wide significance threshold, in view of *ANGPT1*’*s* relevant biological function, we pursued a replication of this signal in a larger aSAH patient cohort.

The replication cohort was collected during similar periods of time as the discovery cohort (Fig S2). Characteristics of the replication cohort are summarized in Table 1. We assayed 58 discovery samples and 175 replication samples by MethylSeq at the replication stage. The entire cohort (*N* = 233) was majority White (89%) with an age range from 25 to 75 and a female proportion of 72%. Following the aSAH, 80 participants developed DCI and 153 did not. DCI cases and controls were similar in mean age (52.2 and 53.0), female proportion (67.5% and 74.5%), and proportion of smokers (67.6% and 64.0%). We successfully followed up 186 participants at 3 months post-discharge, 20 of whom had died; likewise, 115 were reached at 12 months and 24 died. The discovery and replication subsets were similar with respect to the demographic and clinical characteristics, except that the discovery group had a significantly higher DCI frequency (*p*-value = 0.02), as we intentionally balanced the number of DCI cases and controls when selecting samples to be measured by the Illumina 450k Beadchip.

**Fig. 2:**
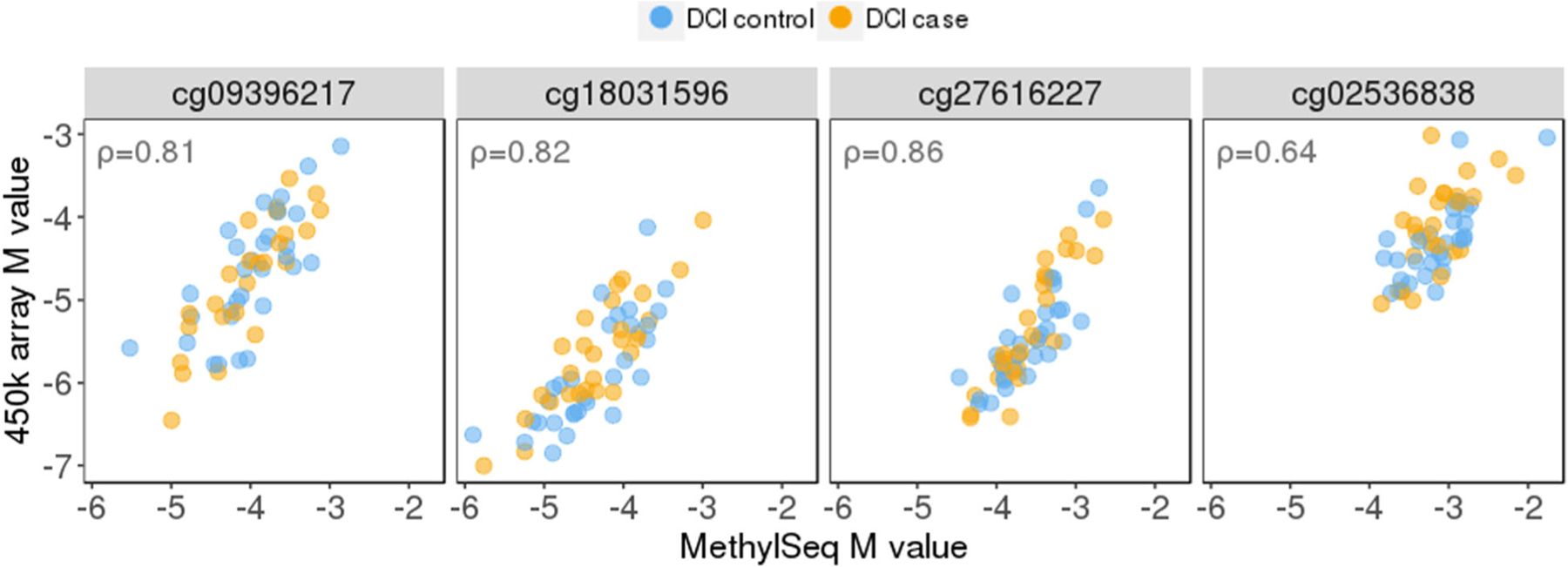
Correlations between the 450k array M values and the MethylSeq M values at four CpG sites among 58 discovery samples with both measurements available. The Spearman correlation coefficients are shown in the upper left of each sub-figure. Colored by DCI status

We used MethylSeq to assay methylation levels at five CpG sites in the *ANGPT1* region. The mean percentage of methylated calls at these targeted sites ranged from 5.24 to 10.85%. The five targeted CpGs were correlated with each other with pairwise correlation coefficients *ρ* ranging from 0.69 to 0.86 (Fig S3). Four out of the five CpGs were also measured by the 450K array (chr8: 108510324 was not). When we compared the 450K and the MethylSeq methylation measures among the 58 discovery samples assayed in both stages, we observed high correlations between the *M* values (Fig. 2).

To replicate the signal at the *ANGPT1* locus, we first analyzed the 175 replication samples (which do not overlap with the initial discovery cohort). Four out of the five CpGs had a *p* value below the Bonferroni adjusted threshold (*p*-value < 0.045) (Table 2). The EWAS top CpG cg18031596, with a replication *p*-value = 0.021, had a regression coefficient of −0.200 (95% CI −0.369, −0.030), indicating that its methylation level was on average lower in DCI cases (mean beta-value = 4.84%) than in DCI controls (mean beta-value = 5.56%). The average methylation level measured as beta-values for all five sites are shown in Table S4. The direction of effect in the replication sample was consistent across all five sites (all had negative coefficients), which is expected given the high degree of correlation among them. In the combined sample (discovery and replication together, *N* = 233), three out of five sites were significant, and the CpG at chr8:108510324, which was not measured in the discovery EWAS analysis, yielded the smallest *p*-value = 0.004. We noticed attenuated effect sizes of all CpGs when adding in the 58 samples from the discovery stage. For cg18031596, its negative effect direction in the non-overlapping replication sample or the combined sample contrasts with the result based on the discovery sample alone, where it displayed a positive effect direction, despite the *p*-value being not significant (coefficient = 0.102, *p*-value = 0.52; Table S5). The other four CpGs had consistent negative effect directions in the analyses based on discovery sample alone (Table S5) and in the analyses based on replication sample alone (Table 2). To confirm that the smoking status was not confounding our results, we also performed the replication association tests while additionally adjusting for smoking, and the results were largely similar (Table S6).

**Fig. 3:**
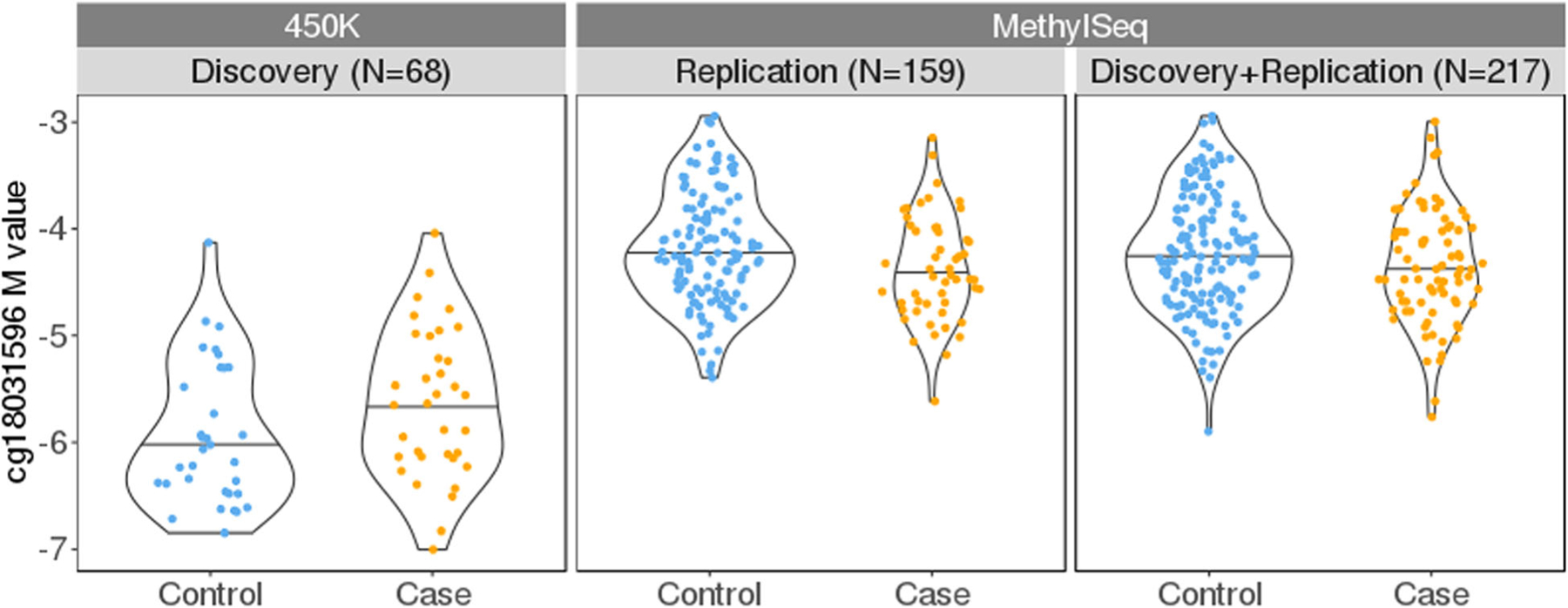
Opposite effect directions of cg18031596 in the discovery and replication analysis. The horizontal line segment represents the median. DCI cases on average had a higher *M* value than controls in the discovery stage, yet in the replication samples the opposite was observed

The “450K” and “MethylSeq” columns in Table 2 showed conflicting signs of the regression coefficients of *ANGPT1* CpGs, i.e., higher levels of *ANGPT1* methylation were associated with an increased risk of DCI in the discovery EWAS yet with a decreased risk in the replication analysis. This discrepancy in the association directions can be visualized in Fig. 3 with cg18031596 as an example (Fig S4 for the other four sites), and Fig S5 further displays smokers and non-smokers separately. We asked if the difference in whether or not surrogate variables are used can lead to the observed inconsistent effect directions. As shown in Table S5, analysis adjusting/not adjusting for proxy surrogate variables did give opposite directions of effect for three CpG sites but not for cg18031596, although none of the *p*-values in this small subset of samples were less than 0.05.

In the analysis of recovery outcomes using the Methyl-Seq data, a higher *M* value at cg18031596 was associated with a lower risk of death at 3 months, with a *p*-value = 0.029 (Table S7). The suggestive associations for cg18031596 with GOS-3 and GOS-12 from the discovery analysis did not show evidence of replication.

### Simulation results

**Fig. 4:**
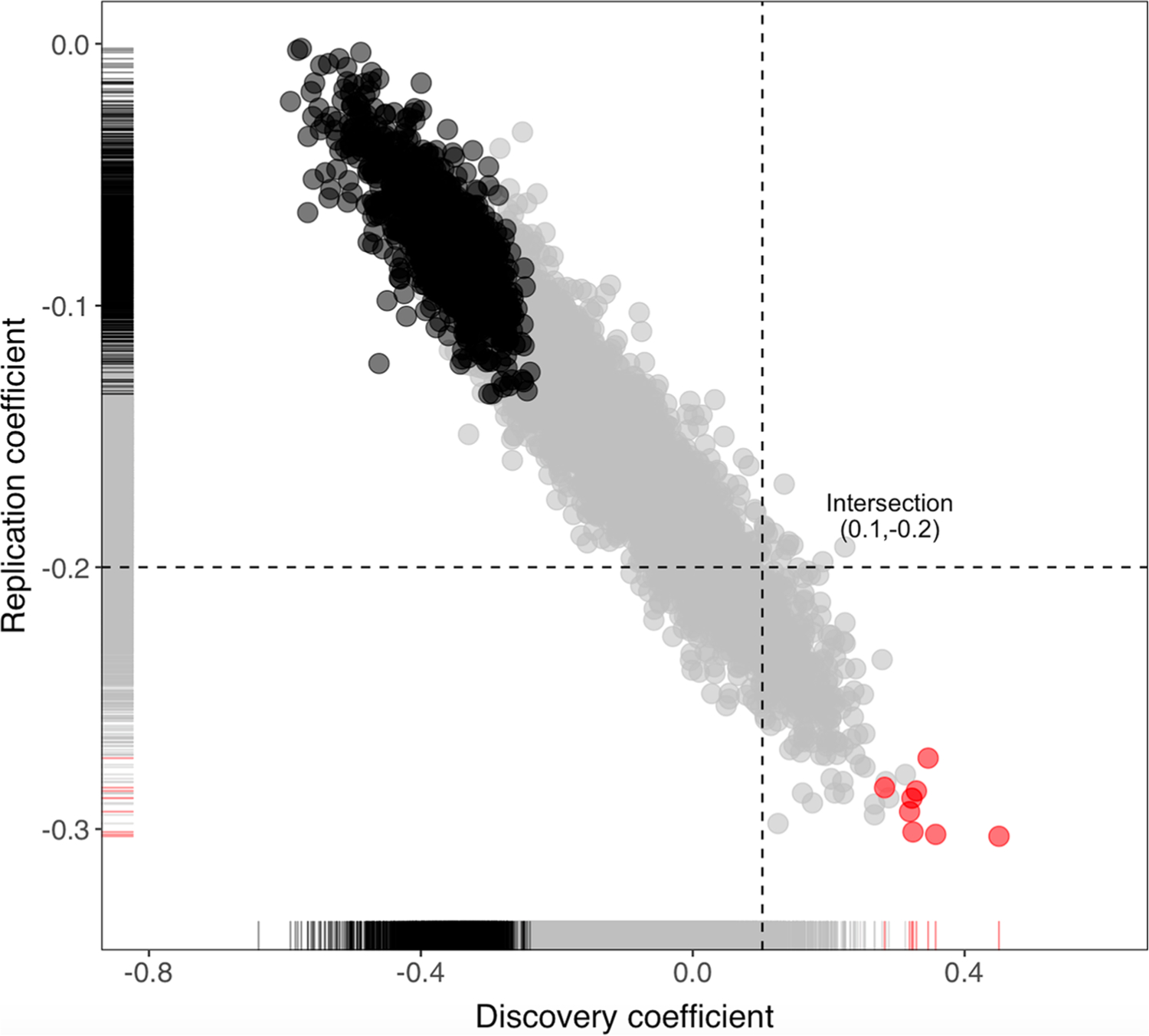
Regression coefficients in simulated discovery (*x*-axis) and replication (*y*-axis) samples. Results from 10,000 simulation experiments are plotted. Black and red dots mark the 1229 samplings which yielded a discovery *p*-value < 0.05; the background gray dots represent the remaining non-significant ones (*p*-value > 0.05). Coefficients were derived from a model where *M* value at cg18031596 was regressed on DCI and covariates. Marginal distributions are indicated by the rug plots along the inner side of the axes. The horizontal and vertical dashed line identify the observed replication coefficient (−0.2) and observed discovery coefficient (0.1; can be found in Table S3), respectively. Red dots represent a flip of association direction with the simulated replication coefficient more extreme than the observed replication coefficient

The flip in effect direction at cg18031596 between the discovery and replication results may have occurred by random chance. We estimated the likelihood of obtaining two significant but opposite associations due to random chance by randomly splitting all samples assayed by MethylSeq (*N* = 233) into two sets, one of size *N* = 58 (“discovery”) and the other of size *N* = 175 (“replication”). These two sets were then tested separately for the association between DCI and methylation at the top EWAS CpG cg18031596 in the same way as we did for the real data. Among the 10,000 simulations, 1229 of them yielded a discovery *p* value < 0.05 (mimicking the small EWAS *p*-value which led us to a replication), and among these 1229 significant discovery samplings, the effect sizes in eight samplings were farther away from null in the positive direction than the observed effect of cg18031596 in real data. This is shown in Fig. 4 where eight red dots fall farther to the right of the vertical line indicating the magnitude of the observed coefficient in the real discovery sample. These eight samplings had positive discovery coefficients but negative replication coefficients. Their replication *p*-values ranged from 2.8 × 10^−4^ to 0.0017, all being significant according to our replication significance threshold (*p* < 0.045), indicating a successful replication with the sign of the coefficient flipped. That said, it would be very likely (8 out of 8) to observe a direction-flipped association, once the discovery sample by chance yielded a significant positive association as extreme or more extreme than the observed one, although the precondition is a rare chance (8 out of 1229). The discovery and replication coefficients had a Pearson correlation of −0.87 among 1229 simulations, demonstrating a high level of dependency between the discovery results and the replication results under the simulated settings.

## Discussion

In this study, we examined the role of epigenome-wide DNA methylation profiles in DCI occurrence and recovery outcomes among aSAH patients. None of the CpG sites passed the EWAS significance threshold of 2.4 × 10^−7^. The top hit at cg18031596 had a small *p*-value of 2.3 × 10^−6^ and was annotated to *ANGPT1*, which is a key player in angiogenesis after vascular injury. Despite having a small cohort, at cg18031596 we estimated a 94.6% power to detect a true mean difference as larger or larger than the observed mean difference at a significance threshold of *p*-value =1 × 10^−7^. With an intention not to miss a potentially true signal with biological relevance, we conducted targeted bisulfite sequencing of *ANGPT1* in a larger follow-up patient cohort. Although four out of the five CpG sites sequenced were significantly associated at a Bonferroni adjusted threshold, their effects were in the opposite direction compared to the EWAS results. Our mixed results indicate that more research is needed to determine the relevance of ANGP T1 for the occurrence of DCI.

The top EWAS CpG site, cg18031596, is located immediately upstream the transcription start site of *ANGP T1* (Fig. 2C). *ANGPT1* encodes angiopoietin-1 (ANG-1), which belongs to the angiopoietin family and plays important roles in vascular development and angiogenesis. Studies have shown that by acting through Tie2, an endothelial-specific tyrosine kinase receptor, ANG-1 mediates the endothelium-surrounding matrix interactions, maintains the integrity of the vascular endothelium, and inhibits endothelial permeability to protect blood vessels from leaking in vivo [19–21]. ANG-1 was also known as an anti-inflammatory factor, and inflammation was actually one of the proposed processes involved in the development of DCI [8, 9, 22]. Mice overexpressing Ang-1 displayed greater resistance to leaks caused by inflammatory agents and a reduced ischemic lesion volume after embolic middle cerebral artery occlusion [23, 24]. Several preclinical studies have shown a therapeutic effects of ANG-1 in alleviating the consequence of ischemia and stroke [25–27]. In a previous longitudinal study, decreased serum levels of ANG-1 early after admission due to aSAH was found in patients who suffered DCI later, and these patients experienced a delayed increase of ANG-1 compared with DCI controls [28]. Polymorphisms in the mice ortholog of *ANGPT1* were reported as genome-wide significant hits in a study of mice using cerebral artery occlusion model, and human population studies have reported a few genetic variants of *ANGPT1* associated with ischemic and hemorrhagic stroke [29, 30]. As the relationship between DNA methylation and gene expression is tissue-specific and dynamic, it is so far not known if the methylation status of cg18031596 indeed regulates the expression level of *ANGPT1* in DCI occurrence. By querying a public dataset containing methylation and mRNA data, we found that there was a correlation between cg18031596 and *ANGPT1* expression in several tissues (TCGA Wanderer dataset, http://maplab.imppc.org/wanderer/)) (Fig S6). Future studies are needed to establish the regulatory effect of methylation at cg18031596 on the expression of *ANGPT1*.

We note that we were not able to rule out the potential confounding effect from factors known to influence methylation level, such as existing cerebrovascular and cardiovascular conditions of the patients, certain genetic variants and environmental stimuli [31, 32]. The lack of the genotype data of the studied patients prevented us from identifying and controlling for methylation quantitative trait loci (meQTL) as common causes for DCI and methylation. As the current knowledge on DCI etiology is far from complete, factors that have not been studied and recognized in the context of DCI also have the potential to confound our results in addition to known DCI risk factors. Replications in larger independent cohorts with more complete data are in need to further follow-up our findings.

Although the post hoc power calculation for the top EWAS hit cg18031596 indicated an adequately powered discovery analysis, the failure in replicating this signal suggests that it was likely a random chance that *ANGP T1* ranked top in the EWAS and this did not represent a true signal. In addition to this explanation, we performed several examinations to explore other possibilities for the discordance in effect directions. Some typical reasons for a flip in association directions include differences in methylation correlation patterns across populations, a lack of power in the smaller study, sampling variation, and different genomic and/or environmental context. Our discovery and replication samples were largely of the same ethnic origins (Table 1) so we did not expect them to differ much in methylation correlation patterns. Other possibilities were discussed in the following paragraphs.

First, this inconsistency may be attributed to a complex interplay among disease-influencing factors coupled by differential distribution of these factors in the discovery and replication samples. We sought to investigate this possibility by examining available demographic and clinical variables of the participants. We did not observe significant differences in the distribution of demographics (age, sex, race, height, weight) nor the clinical characteristics (Fisher score, recovery outcomes) between the discovery and the replication groups (Table 1). We noticed remarkably high smoking rates among the participants from both groups, in line with smoking being a risk factor for aSAH. Although not statistically significant, the replication group had more smokers than the discovery group did (Table 1, 68% vs 57%, fisher exact test *p* = 0.14). Smoking is a well-established trigger of methylation signature alteration [33], and there have been studies reporting an alteration of *ANGPT1* methylation or ANG-1 protein levels in samples from smokers compared with non-smokers [34, 35]. We explored whether smoking was able to complicate the analysis and contributed to the effect direction flip, and observed a complicated influence of smoking as some CpGs showed a flip in direction only in smokers while others had a flip in direction only in non-smokers (Fig S5). However, given the small group sizes, we were not able to make any conclusion. Although we used surrogate variables in the EWAS to control for unwanted variations not explicitly adjusted as a covariate, such as smoking status, it is possible that smoking status may have complicated our results in a way that was not easy to unmask through the inspections we could perform given the amount of data available. We found that the effect direction discrepancy could not be explained by race, time of DNA collection, or Fisher score. It is possible that complex interactions between either measured or unmeasured factors are in part responsible for the reversed directions, even though we were not able to statistically detect the effect.

One major difference between the discovery and replication analyses was the technology used for methylation profiling. Although *ANGPT1* methylation levels quantified by 450K array and MethylSeq showed medium to high correlation (*ρ* = 0.64~0.86, Fig. 2), there was certainly some degree of inconsistency. Indeed, the *M* values were far from being identical. This difference in methylation measurements may contribute to the flip in effect direction in the first place.

We then speculated that the adjustment for surrogate variables in the discovery EWAS and the inability of doing so in the replication cohort may give rise to effect estimates of opposite directions. The results from the proxy surrogate variable analyses support that this could account for the sign flip at four of the five *ANGPT1* CpGs but not at cg18031596 (Table S5).

Lastly, we explored whether the discrepancies in effect directions of cg18031596 could be explained by chance, provided that the association is not a false positive. Considering the entire sample, if the discovery EWAS by chance oversampled from the upper tail of the methylation distribution of DCI cases and the lower tail of the methylation distribution of DCI controls, then the remaining samples left for the replication analysis would give the impression of an effect in the opposite direction. This explanation was supported by our simulation experiments where the chance of such oversampling was 8 in 1229. Sampling variation like this is unlikely but not impossible, and once this has happened, an opposite effect estimate is highly likely to follow (100% of the time in our simulation). A sample size as small as a few dozen added an extra possibility of large statistical fluctuations. It is also possible that the observed opposite effects were the results of a combination of multiple explanations discussed above.

We found some evidence of associations between *ANGPT1* methylation and patient recovery at 3 and 12 months post hemorrhage, although this does not replicate in the follow-up cohort (where we cannot adjust for surrogate variables). This is consistent with a previous study demonstrating that a high concentration of serum ANG-1 predicts better 3-month post-hemorrhage GOS [36]. This relatively long-term effect of *ANGPT1* methylation may be mediated through its effect on DCI risk, since patients displaying higher level of methylation tend not to suffer DCI and therefore recover better. The relationship may also be independent of or only partially mediated by DCI risk, which will need to be examined in future studies.

This study has several strengths. Cross-sectional EWAS is often criticized for the possibility of reverse causality. Although methylation can both influence and be influenced by health conditions, the longitudinal design of our study naturally established the proper temporality. The use of surrogate variables minimized confounding from technical artifacts and cell type heterogeneity. We also note several limitations that should be taken into consideration for result interpretation. First, it is still under debate that if blood is a relevant tissue for studying brain conditions. For a study of DCI, there is some evidence suggesting its usefulness. Immune cell populations are likely to reflect biological changes ensuing inflammatory response, which has been shown to correlate with DCI events [8, 9]. Second, the targeted replication assay limited our ability to adjust for cell type heterogeneity in the replication analysis, which, as discussed above, may contribute to the inconsistent effects of *ANGPT1* methylation in the discovery and the replication analysis. Third, although we excluded any samples collected after the first 48 h post hemorrhage, there may still be some level of heterogeneity among samples within the 48-h period. Fourth, sample size is a recurrent challenge in studies of diseases with low incidence and high mortality, and we were not able to seek external replication cohorts with DNA methylation data collected. One prominent consequence of the small sample size is that the statistical model may not sustain an adjustment of all necessary covariates, and this prevented us from adjusting for factors that are thought to be clinically important for DCI, such as Fisher scale and smoking status. Nonetheless, we applied surrogate variable analysis which is designed to remove the effect of any uncontrolled variables. Lastly, there were some lost to follow-up at 3 months and 12 months post injury, which may affect our analysis of recovery outcomes at those time points if the drop-out was not random.

## Conclusions

In conclusion, this study did not find robust evidence that *ANGPT1* methylation is associated with DCI occurrence in two aSAH patient cohorts. Given the gene’s relevant function in DCI pathology and its top rank of significance in the EWAS, our results call for future studies of larger aSAH cohorts to determine its relevance in the occurrence of DCI.

## Supplementary Material

1783375_Sup_material **Additional file 1**. Supplementary Methods. Supplementary Table S1–S7. Supplementary Figure S1–S6

## Figures and Tables

**Table 1:** Characteristics of the study population

	450K	MethylSeq (*N* = 233)		
	Discovery (*N* = 68)	Discovery (*N* = 58)	Replication (*N* = 175)	*p*-value^a^
Demographic				
Age (years)	53.7 ± 11.3	53.3 ± 11.2	52.5 ± 11.0	0.66
Sex (female/male)	48/20 (71%)	39/19 (67%)	129/46 (74%)	0.40
Race (white/black)	57/9 (86%)	50/8 (86%)	154/17 (90%)	0.47
Smoke (yes/no)	35/25 (58%)	30/23 (57%)	107/50 (68%)	0.14
Clinical				
Fisher (2/3/4)	17 (26%)/34 (51%)/15 (23%)	16 (28%)/32 (55%)/10 (17%)	72 (41%)/79 (45%)/24 (14%)	0.19
DCI (yes/no)	35/33 (51%)	28/30 (48%)	52/123 (30%)	0.02
Time of DCI (days post injury)	6.5 ± 2.5	6.4 ± 2.3	5.8 ± 2.3	0.22
GOS-3 (unfavorable/favorable)	19/34 (36%)	13/31 (30%)	27/115 (19%)	0.15
GOS-12 (unfavorable/favorable)	11/38 (22%)	7/34 (17%)	20/54 (27%)	0.26
MRS-3 (unfavorable/favorable)	35/18 (66%)	27/17 (61%)	71/71 (50%)	0.23
MRS-12 (unfavorable/favorable)	24/25 (49%)	18/23 (44%)	34/40 (46%)	0.85
Death-3 (dead/alive)	5/48 (9%)	3/41 (7%)	17/125 (12%)	0.42
Death-12 (dead/alive)	9/40 (18%)	6/35 (15%)	18/56 (24%)	0.24

Variable values are presented as mean ± sd for continuous variables, category count followed by percentage of the first category for binary variables, and count (percentage) for multi-categorical variables. The discrepancy between count sum and sample size was a result of missingness in observations

a Characteristics of the MethylSeq discovery (*N* = 58) and replication sample (*N* = 175) were compared by *t*-test or Fisher’s exact test

**Table 2:** Association results of *ANGPT1* methylation and DCI in the EWAS (450K) and the replication (MethylSeq) analyses

CpG site	Position^a^	450K			MethylSeq					
		Discovery^b^ (*N* = 68)			Replication (*N* = 175)			Discovery + replication (*N* = 233)		

		Coefficient	95% CI	*p*-value	Coefficient^c^	95% CI	*p*-value^d^	Coefficient^c^	95% CI	*p-*value^d^
cg09396217	chr8:108510286	0.044	− 0.135, 0.222	0.63	− 0.215	− 0.389, − 0.040	**0.016**	− 0.185	− 0.333, − 0.038	**0.014**
cg18031596	chr8:108510292	0.398	0.244, 0.552	3.35 × 10^−6^	− 0.200	− 0.369, − 0.030	**0.021**	− 0.145	− 0.293, 0.003	0.054
cg27616227	chr8:108510314	0.189	0.009, 0.368	0.04	− 0.149	− 0.290, − 0.009	**0.038**	− 0.123	− 0.240, − 0.006	**0.040**
chr8: 108510324	chr8:108510324	-		-	− 0.177	− 0.327, − 0.027	**0.021**	− 0.176	− 0.297, − 0.056	**0.004**
cg02536838	chr8:108510343	0.301	0.147, 0.455	2.52 × 10^−4^	− 0.116	− 0.242, 0.011	0.073	− 0.097	− 0.205, 0.011	0.077

a GRCh37.p13

b Model M ~ DCI + age + sex + surrogate variables. The coefficients, CIs, and *p*-values under the “450K” meta-column are different from the EWAS results because we used a different statistical method for model parameter estimation in order to be consistent with the method used for analyzing the MethylSeq data (those shown under the “MethylSeq” meta-column). The purpose was to make the results more directly comparable

c Model M ~ DCI + age + sex. Represents the average difference in *M* value between cases and controls, with control being the reference

d Bold *p*-values are less than the Bonferroni significance threshold for replication analysis: *p*-value < 0.05/1.1 = 0.045
