# Supplementary material for: ANGPT1 methylation and delayed cerebral ischemia in aneurysmal subarachnoid hemorrhage patients
Source: Epigenetics Commun. Author manuscript; Available in PMC 2022 Mar 30. (PMC8967216; doi:10.1186/s43682-021-00001-7)

**SUPPLEMENTARY MATERIALS**

*ANGPT1* methylation and delayed cerebral ischemia in aneurysmal subarachnoid hemorrhage patients

Dongjing Liu^1^, Annie I. Arockiaraj^2^, John R. Shaffer^2,3^, Samuel M. Poloyac^4^, Paula R. Sherwood^5^, Sheila A. Alexander^5^, Elizabeth A. Crago^5^, Daniel E. Weeks^2,6^, Yvette P. Conley^2,5^

Correspondence:

Yvette P. Conley

yconley@pitt.edu

Supplemental Methods

Targeted MethylSeq probe info

NC_000008.10 Homo sapiens chromosome 8, GRCh37.p13 Primary Assembly

chr8:108510282-108510356

GYRGCGGGATCGTCTTACAAATTGGCTAGATGCGCATGACCTCGATCATGTATGGCCCTCCCGCCCCTTTCCACA

The five variable CpG sites are highlighted.

Table S1. CpG probe-level filters and the number of probes removed at each step

| **Sequential filtering step** | **Filtered** | **Retained** |
| --- | --- | --- |
| none |  | 485,512 |
| probe sequence overlapping SNP | 17,541 | 467,971 |
| off-target cross-reactivity | 36,489 | 431,482 |
| sex chromosome | 10,191 | 421,291 |
| multimodal beta-value distribution | 2,072 | 419,219 |
| low-quality detection | 972 | 418,247 |

Table S2. Top 10 CpGs from the discovery EWAS

| CpG site | Position* | Gene | Log fold change** | Mean methylation beta-value in controls | Mean methylation beta-value in cases | *p-*value | Bayes factor |
| --- | --- | --- | --- | --- | --- | --- | --- |
| cg18031596 | chr8:108510292 | *ANGPT1* | 0.40 | 1.79% | 2.20% | 2.28E-06 | 2.44 |
| cg07875082 | chr16:2795390 | *LOC100128788* | 0.28 | 87.54% | 89.13% | 7.97E-06 | 1.66 |
| cg26570279 | chr16:58324876 | *KLKBL4* | -0.33 | 88.90% | 86.72% | 1.12E-05 | 1.45 |
| cg07146531 | chr1:187343088 | *-* | -0.38 | 77.71% | 73.57% | 1.12E-05 | 1.45 |
| cg06627361 | chr12:498468 | *KDM5A* | 0.22 | 0.48% | 0.57% | 1.20E-05 | 1.41 |
| cg14691388 | chr8:140711270 | *KCNK9* | -0.32 | 81.00% | 78.11% | 1.39E-05 | 1.31 |
| cg26979473 | chr7:137028389 | *PTN* | -0.32 | 4.43% | 3.97% | 2.24E-05 | 1.02 |
| cg19138227 | chr17:42809833 | *DBF4B* | 0.33 | 80.62% | 84.08% | 2.42E-05 | 0.97 |
| cg17783015 | chr6:161258752 | *-* | -0.31 | 61.08% | 54.99% | 2.62E-05 | 0.92 |
| cg09071459 | chr16:4903054 | *UBN1* | 0.29 | 86.55% | 88.09% | 3.39E-05 | 0.76 |

* GRCh37.p13

** Log fold change represents the fold change of M value in the cases compared with the controls in the log scale. Obtained by regressing M value against DCI status while adjusting for surrogate variables and age and sex.

Table S3. Association results of the methylation level at *ANGPT1* CpG sites and recovery outcomes in the discovery (EWAS) sample

| Recovery outcome | CpG site | Log fold change in M-values* | Mean beta-value in the favorable group | Mean beta-value in the unfavorable group | *p* |
| --- | --- | --- | --- | --- | --- |
| GOS at 3 months | cg09396217 | 0.044 | 4.19% | 3.74% | 0.698 |
|  | cg18031596 | 0.292 | 1.93% | 2.15% | 0.006 |
|  | cg27616227 | 0.149 | 2.63% | 2.54% | 0.177 |
|  | cg02536838 | 0.196 | 5.58% | 5.62% | 0.067 |
| GOS at 12 months | cg09396217 | 0.343 | 4.31% | 4.02% | 0.010 |
|  | cg18031596 | 0.285 | 2.08% | 2.21% | 0.047 |
|  | cg27616227 | 0.173 | 2.83% | 2.59% | 0.236 |
|  | cg02536838 | 0.251 | 5.70% | 5.70% | 0.053 |
| MRS at 3 months | cg09396217 | 0.218 | 3.67% | 4.22% | 0.049 |
|  | cg18031596 | 0.172 | 1.86% | 2.08% | 0.114 |
|  | cg27616227 | 0.174 | 2.55% | 2.62% | 0.113 |
|  | cg02536838 | 0.242 | 5.04% | 5.89% | 0.022 |
| MRS at 12 months | cg09396217 | 0.182 | 4.41% | 4.07% | 0.082 |
|  | cg18031596 | 0.272 | 2.13% | 2.09% | 0.012 |
|  | cg27616227 | 0.123 | 2.90% | 2.65% | 0.274 |
|  | cg02536838 | 0.282 | 5.52% | 5.89% | 0.004 |
| Death at 3 months | cg09396217 | 0.190 | 4.05% | 3.83% | 0.351 |
|  | cg18031596 | 0.223 | 2.00% | 2.12% | 0.260 |
|  | cg27616227 | 0.372 | 2.61% | 2.46% | 0.058 |
|  | cg02536838 | 0.168 | 5.67% | 4.96% | 0.390 |
| Death at 12 months | cg09396217 | 0.284 | 4.29% | 4.02% | 0.052 |
|  | cg18031596 | 0.166 | 2.11% | 2.10% | 0.291 |
|  | cg27616227 | 0.172 | 2.82% | 2.59% | 0.275 |
|  | cg02536838 | 0.209 | 5.76% | 5.45% | 0.140 |

* Log fold change represents the fold change of M value in the unfavorable group compared with the favorable group in the log scale. Obtained by regressing M value against recovery outcomes while adjusting for 5-7 surrogate variables plus covariates.

Table S4. Mean methylation beta-values in controls and cases, corresponds to the comparisons in Table 2.

| CpG site | Position | 450K | |  | MethylSeq | | | | |
| --- | --- | --- | --- | --- | --- | --- | --- | --- | --- |
|  |  | Discovery (N = 68) | |  | Replication (N = 175) | |  | Discovery + Replication (N = 233) | |
|  |  | Control | Case |  | Control | Case |  | Control | Case |
| cg09396217 | chr8:108510286 | 4.05% | 4.10% |  | 7.07% | 5.99% |  | 6.90% | 6.04% |
| cg18031596 | chr8:108510292 | 1.79% | 2.20% |  | 5.56% | 4.84% |  | 5.41% | 4.93% |
| cg27616227 | chr8:108510314 | 2.43% | 2.76% |  | 8.52% | 7.64% |  | 8.40% | 7.74% |
| chr8:108510324 | chr8:108510324 | - | - |  | 6.07% | 5.30% |  | 5.99% | 5.25% |
| cg02536838 | chr8:108510343 | 4.99% | 5.93% |  | 11.19% | 10.49% |  | 11.05% | 10.48% |

Table S5. Association results of the methylation level at *ANGPT1* CpG sites and DCI in the MethylSeq discovery sample (N=58), with/without adjustment for proxy surrogate variables

|  |  | Without sv adjustment | | | With sv adjustment | | |
| --- | --- | --- | --- | --- | --- | --- | --- |
| CpG site | Position | Coefficient* | 95% CI | *p* | Coefficient** | 95% CI | *p* |
| cg09396217 | chr8:108510286 | -0.025 | -0.330, 0.280 | 0.872 | 0.034 | -0.153,0.221 | 0.718 |
| cg18031596 | chr8:108510292 | 0.102 | -0.218, 0.423 | 0.524 | 0.118 | -0.087,0.323 | 0.251 |
| cg27616227 | chr8:108510314 | -0.012 | -0.246, 0.223 | 0.920 | 0.055 | -0.083,0.193 | 0.425 |
| chr8:108510324 | chr8:108510324 | -0.108 | -0.322, 0.106 | 0.316 | -0.105 | -0.298,0.088 | 0.277 |
| cg02536838 | chr8:108510343 | -0.017 | -0.241, 0.207 | 0.881 | 0.048 | -0.094,0.189 | 0.502 |

* Coefficient represents the average difference in M value between cases and controls, with control being the reference, after adjusting for age and gender.

** Coefficient represents the average difference in M value between cases and controls, with control being the reference, after adjusting for age, gender and the surrogate variables derived from the 450K data.

Table S6. Replication analysis results, additionally adjusting for smoking status

|  | MethylSeq | | | | | | |
| --- | --- | --- | --- | --- | --- | --- | --- |
|  | Replication (N = 175) | | |  | Discovery + Replication (N = 233) | | |
| CpG site | Coefficient^a^ | 95% CI | *p*-value^b^ |  | Coefficient^a^ | 95% CI | *p-* value^b^ |
| cg09396217 | -0.198 | -0.047, 0.481 | **0.039** |  | -0.187 | -0.262, 0.008 | **0.018** |
| cg18031596 | -0.169 | -0.385, -0.01 | 0.069 |  | -0.130 | -0.343, -0.032 | 0.099 |
| cg27616227 | -0.135 | -0.35, 0.013 | 0.077 |  | -0.122 | -0.285, 0.025 | 0.052 |
| chr8:108510324 | -0.180 | -0.286, 0.015 | **0.024** |  | -0.177 | -0.244, 0.001 | **0.006** |
| cg02536838 | -0.127 | -0.336, -0.024 | 0.066 |  | -0.100 | -0.302, -0.052 | 0.084 |

^a^ Model M ~ DCI + age + sex + smoking. Represents the average difference in M value between cases and controls, with control being the reference.

^b^ Bold p-values are less than the Bonferroni significance threshold for replication analysis: p-value < 0.05/1.1=0.045

Table S7. Association results of the methylation level at *ANGPT1* CpG sites and recovery outcomes in all (discovery + replication) samples assayed by MethylSeq

| Recovery outcome | CpG site | Coefficient* | Mean beta-value in the favorable group | Mean beta-value in the unfavorable group | *p* |
| --- | --- | --- | --- | --- | --- |
| GOS at 3 months | cg09396217 | -0.123 | 6.76% | 6.06% | 0.215 |
|  | cg18031596 | -0.151 | 5.42% | 4.80% | 0.132 |
|  | cg27616227 | -0.058 | 8.30% | 7.79% | 0.461 |
|  | chr8:108510324 | -0.009 | 5.85% | 5.68% | 0.915 |
|  | cg02536838 | -0.098 | 11.09% | 10.09% | 0.172 |
| GOS at 12 months | cg09396217 | 0.083 | 6.30% | 6.45% | 0.508 |
|  | cg18031596 | 0.017 | 5.03% | 5.00% | 0.892 |
|  | cg27616227 | 0.064 | 8.03% | 8.17% | 0.503 |
|  | chr8:108510324 | 0.148 | 5.39% | 5.91% | 0.137 |
|  | cg02536838 | -0.020 | 10.68% | 10.24% | 0.827 |
| MRS at 3 months | cg09396217 | -0.031 | 6.78% | 6.46% | 0.693 |
|  | cg18031596 | -0.036 | 5.42% | 5.16% | 0.659 |
|  | cg27616227 | -0.066 | 8.46% | 7.94% | 0.303 |
|  | chr8:108510324 | 0.020 | 5.83% | 5.79% | 0.772 |
|  | cg02536838 | -0.025 | 11.00% | 10.76% | 0.680 |
| MRS at 12 months | cg09396217 | -0.085 | 6.45% | 6.17% | 0.405 |
|  | cg18031596 | -0.094 | 5.15% | 4.86% | 0.373 |
|  | cg27616227 | -0.017 | 8.11% | 8.01% | 0.832 |
|  | chr8:108510324 | 0.103 | 5.34% | 5.72% | 0.217 |
|  | cg02536838 | -0.046 | 10.74% | 10.36% | 0.551 |
| Death at 3 months | cg09396217 | -0.123 | 6.68% | 6.05% | 0.348 |
|  | cg18031596 | -0.284 | 5.39% | 4.38% | 0.029 |
|  | cg27616227 | -0.056 | 8.24% | 7.72% | 0.586 |
|  | chr8:108510324 | -0.066 | 5.85% | 5.47% | 0.553 |
|  | cg02536838 | -0.106 | 10.99% | 9.96% | 0.257 |
| Death at 12 months | cg09396217 | 0.029 | 6.33% | 6.31% | 0.822 |
|  | cg18031596 | -0.090 | 5.09% | 4.76% | 0.495 |
|  | cg27616227 | -0.014 | 8.12% | 7.85% | 0.889 |
|  | chr8:108510324 | 0.050 | 5.49% | 5.60% | 0.632 |
|  | cg02536838 | -0.046 | 10.71% | 10.09% | 0.625 |

* Coefficient represents the average difference of the M value in the unfavorable group compared with the favorable group. Obtained by regressing M value against recovery outcomes while adjusting for covariates.

Figure S1. Flow chart showing the two-stage study design, where a discovery EWAS was followed by a targeted replication analysis of the top signal in additional samples and a subset of the original cohort.


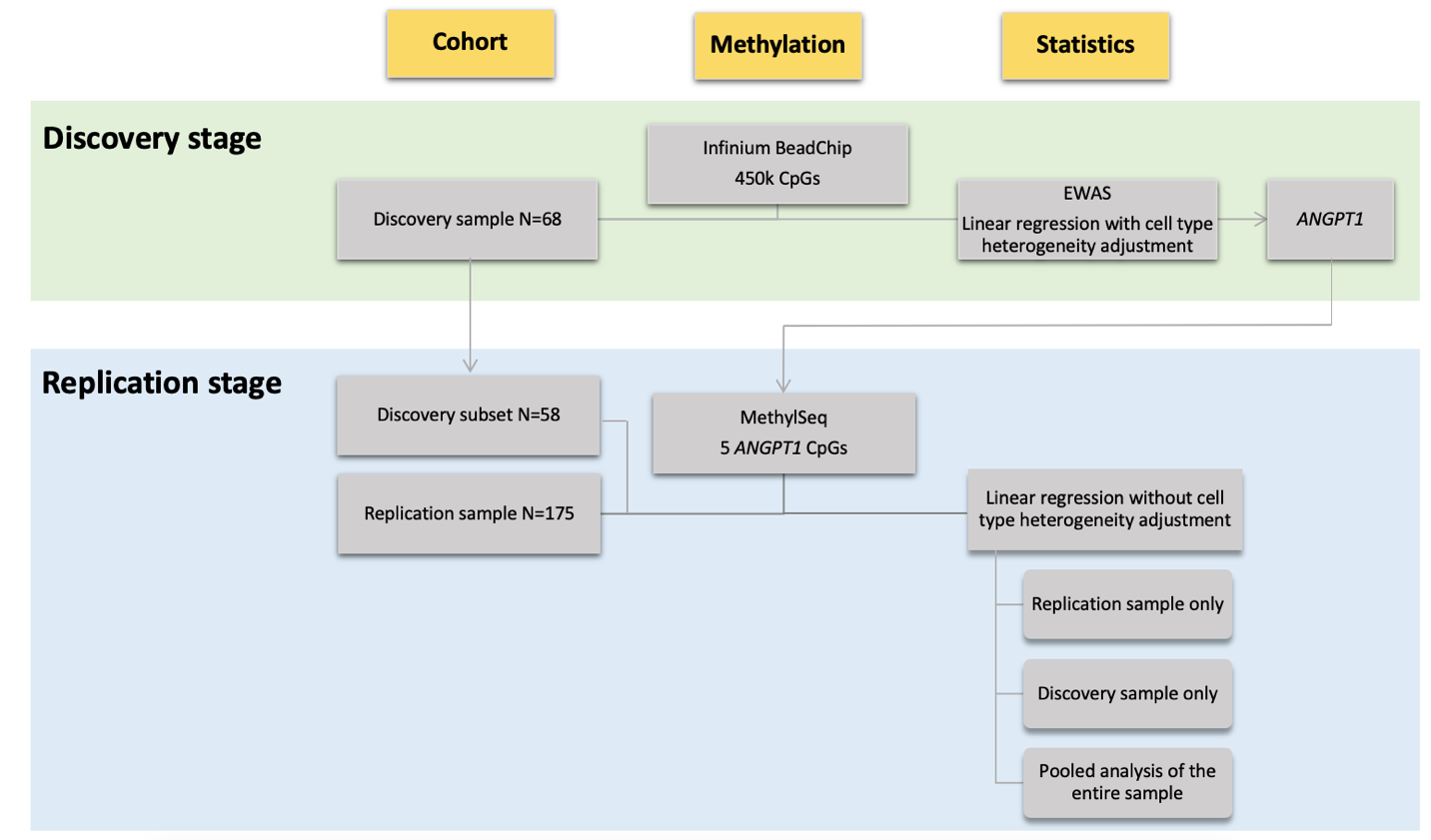


Figure S2. Histogram of the enrollment time of the discovery cohort and replication cohort


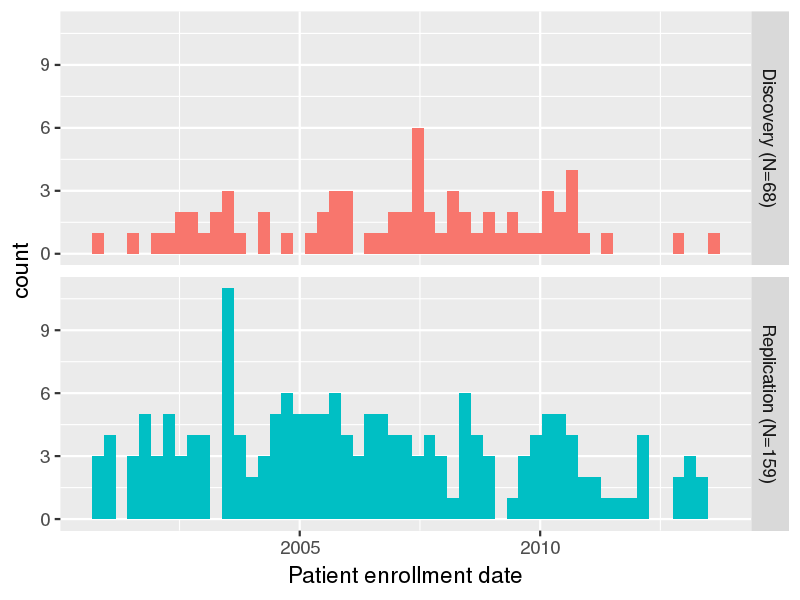


Figure S3. Correlations of the replication methylation levels across five targeted CpG sites


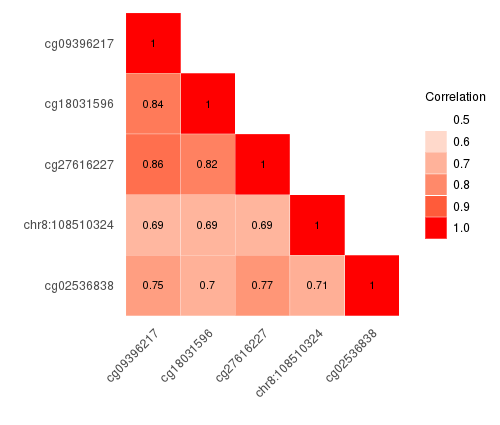


Figure S4. Opposite effect direction of targeted sites (cg09396217, cg27616227, cg02536838, chr8:108510325) in the discovery 450K data (left column) and in the replication MethylSeq data (middle and right columns)


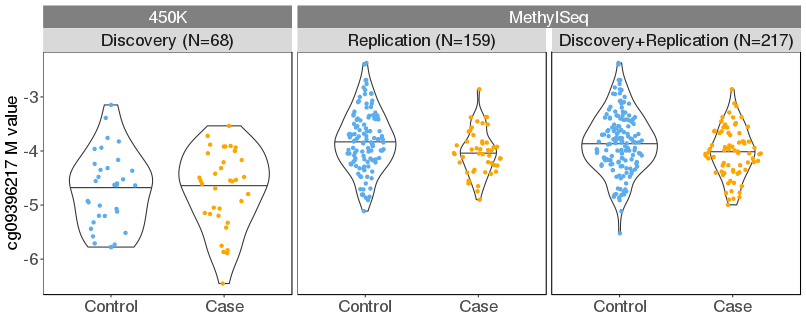

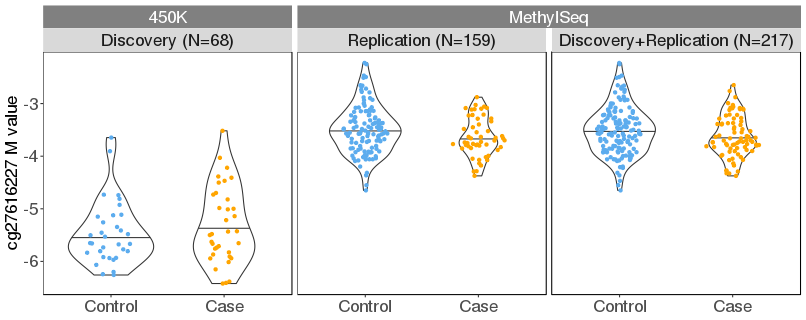


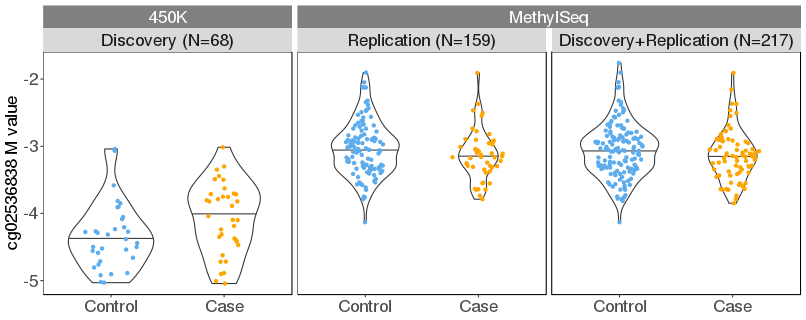


Figure S4 continue


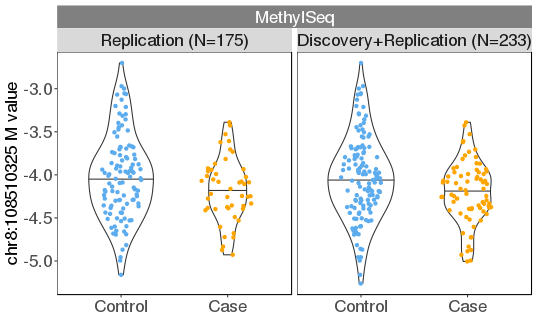


Figure S5. Methylation level by DCI status, in smokers and nonsmokers. Sample sizes in the top row of each subfigure is the maximum possible sample size and may be less due to missingness in smoking status.


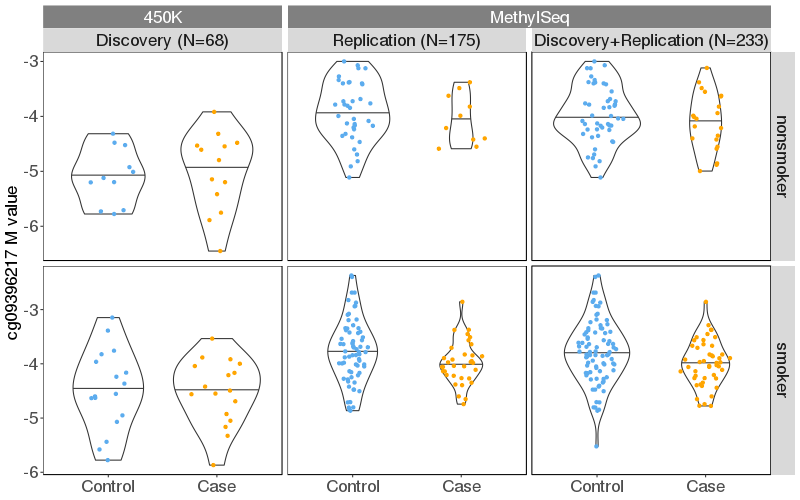


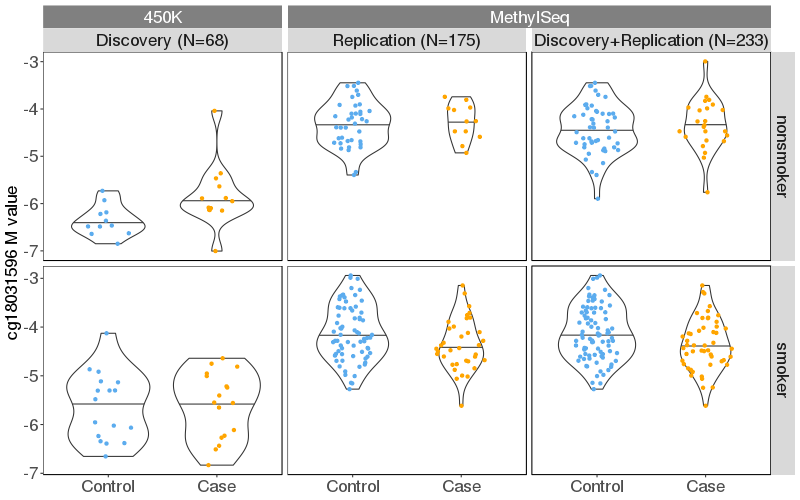


Figure S5 continue


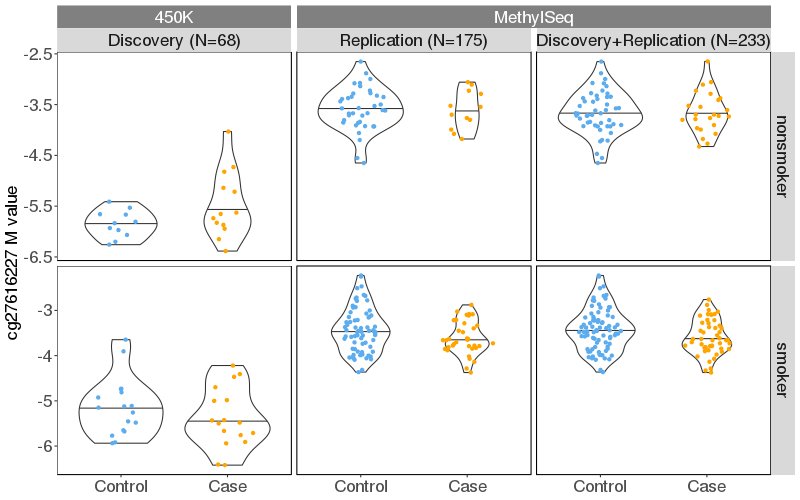


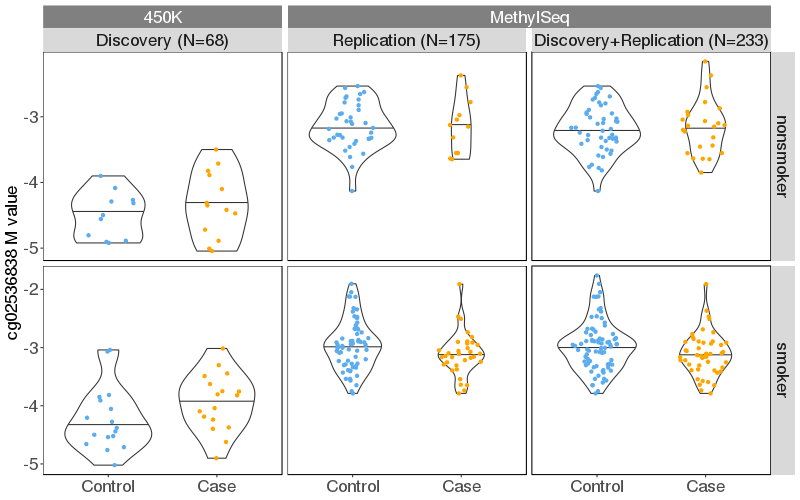


Figure S5 continue


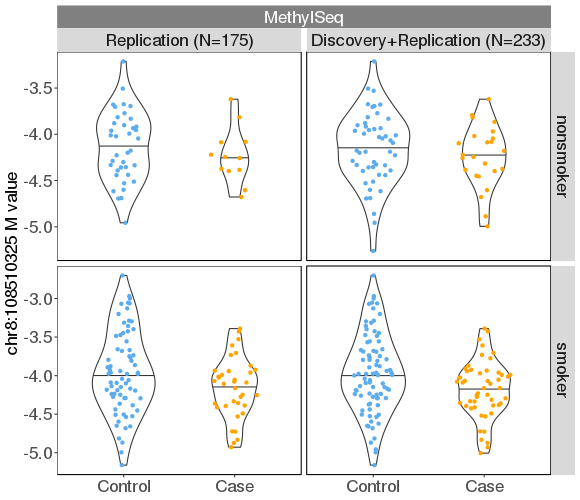


Figure S6. The correlation between cg18031596 methylation and *ANGPT1* expression in normal breast (upper) and thyroid (lower) tissue, from the TCGA Wanderer data viewer. These tissues were chosen as examples to show cg18031596 affects gene expression because they have the largest sample aross all available tissue types.


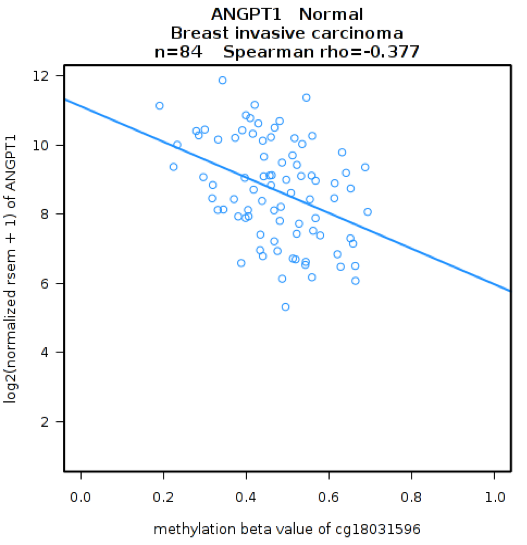


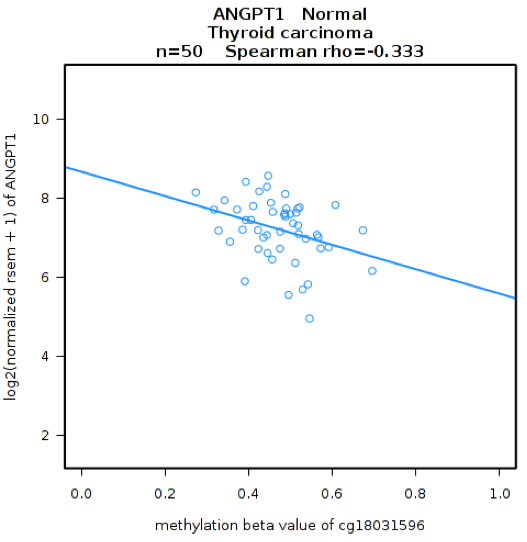

Supplement: 1783375_Sup_material — Additional file 1. Supplementary Methods. Supplementary Table S1–S7. Supplementary Figure S1–S6 [file NIHMS1783375-supplement-1783375_Sup_material.docx]
